# Case Report: A Case Series Using Natural Anatomical Gaps—Posterior Cervical Approach to Skull Base and Upper Craniocervical Meningiomas Without Bone Removal

**DOI:** 10.3389/fsurg.2021.666699

**Published:** 2021-08-17

**Authors:** Nadine Lilla, Almuth F. Kessler, Judith Weiland, Ralf-Ingo Ernestus, Thomas Westermaier

**Affiliations:** ^1^Department of Neurosurgery, University Hospital Wuerzburg, Wuerzburg, Germany; ^2^Department of Neurosurgery, University Hospital Magdeburg, Magdeburg, Germany; ^3^Department of Neurosurgery, Helios-Amper Klinikum Dachau, Dachau, Germany

**Keywords:** minimally invasive, meningioma, cervical spine, spinal tumor operation, craniovertebral junction

## Abstract

**Background:** Removal of anteriorly located tumors of the upper cervical spine and craniovertebral junction (CVJ) is a particular surgical challenge. Extensive approaches are associated with pain, restricted mobility of neck and head and, in case of foramen magnum and clivus tumors, with retraction of brainstem and cerebellum.

**Methods:** Four symptomatic patients underwent resection of anteriorly located upper cervical and lower clivus meningiomas without laminotomy or craniotomy using a minimally invasive posterior approach. Distances of natural gaps between C0/C1, C1/C2, and C2/C3 were measured using preoperative CT scans and intraoperative lateral x-rays.

**Results:** In all patients, safe and complete resection was conducted by the opening of the dura between C0/C1, C1/C2, and C2/C3, respectively. There were no surgical complications. Local pain was reported as very moderate by all patients and postoperative recovery was extremely fast. All tumors had a rather soft consistency, allowing mass reduction prior to removal of the tumor capsule and were well separable from lower cranial nerves and vascular structures.

**Conclusion:** If tumor consistency is appropriate for careful mass reduction before removal of the tumor capsule and if tumor margins are not firmly attached to crucial structures, then upper cervical, foramen magnum, and lower clivus meningiomas can be safely and completely removed through natural gaps in the CVJ region. Both prerequisites usually become clear early during surgery. Thus, this tumor entity may be planned using this minimally invasive approach and may be extended if tumor consistency turns out to be less unfavorable for resection or if crucial structures cannot be easily separated from the tumor.

## Introduction

Anterior and anterolateral tumors of the upper cervical spine, foramen magnum, and clivus are among the most challenging pathologies in neurosurgery. A decisive factor for their successful removal is an appropriate surgical approach. Anterior approaches as well as a variety of posterior approaches have been described to get access to this particular region. The far lateral approach is probably the most extensively used approach for this kind of tumor. However, all these approaches are associated with relevant perioperative morbidity such as nuchal pain, restricted mobility, cerebrospinal fluid (CSF) leakage, manipulation of cranial nerves and/or crossing vessels, and, in some cases, additional stabilization due to postoperative instability (1–3). Minimally invasive approaches to the upper cervical spine and craniovertebral junction (CVJ) have been described (4–7), but they are still rarely reported and/or are rarely used and have been proposed selectively for very small lesions (4).

The posterior aspect of the craniocervical junction exhibits natural bony openings that may be used to access the upper cervical spinal canal, the foramen magnum, and the lower clivus. We report a series of four patients with anterior and anterolateral meningiomas of the foramen magnum, the upper cervical spine, and the lower clivus undergoing complete resection *via* a posterior cervical approach without the removal of any bony structures.

## Methods

Four symptomatic patients with anteriorly located intracranial/intraspinal lesions at the CVJ, which is usually defined from the lower third of the clivus to the vertebral arch of C2 (8), were included in this study. The patients were operated on in our institution between September 2017 and June 2019, and they gave informed written consent in accordance with the local ethic guidelines. We summarized patient data including demographic information, clinical presentation, imaging, operation technique, histology, and the postoperative course.

In all four cases, preoperative imaging (MRI and CT) showed intraspinal and intradural lesions at the CVJ anterior to the medulla oblongata or the upper cervical spinal cord, which were all suspected to be meningiomas. In all cases, complete resection of the tumor was possible, and the patients underwent regular outpatient follow-up.

### Case Descriptions and Surgical Technique

**Figure 1:**
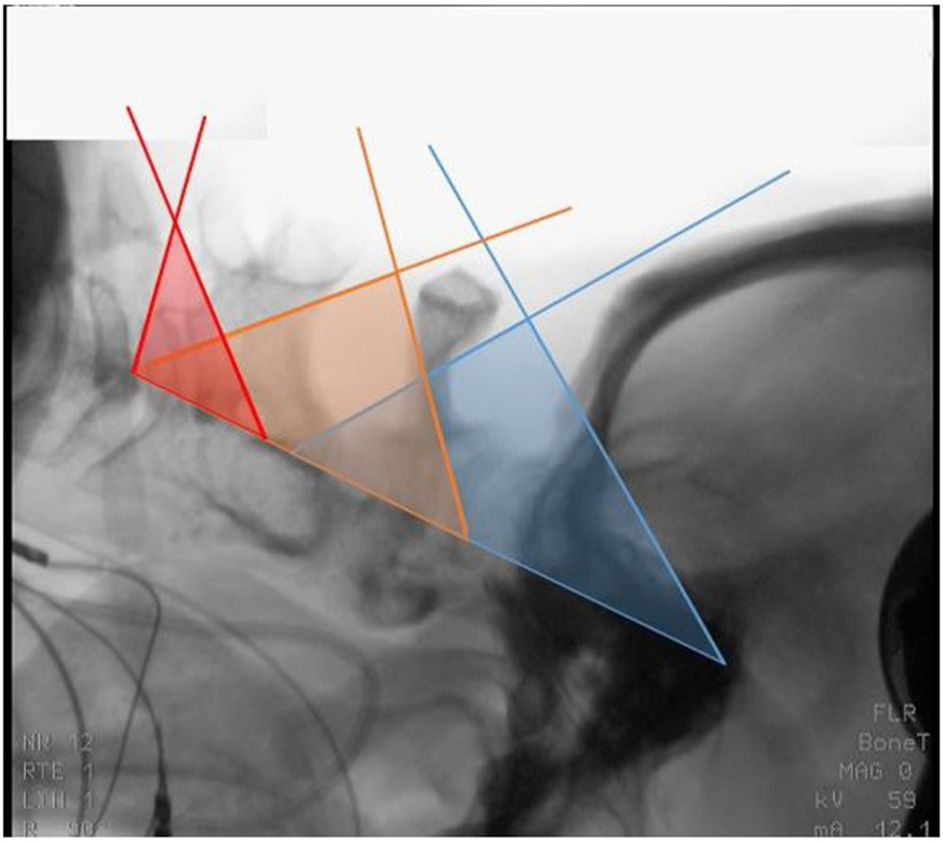
Lateral X-ray of case 4 after prone positioning with inclination. Colored sectors indicate the theoretical access supplied by the interspace between the occiput and the posterior arch of C1 (blue), C1 and the cranial edge of the lamina of C2 (yellow) and, at times, between the inferior edge of the lamina of C2 and the superior edge of the lamina of C3 (red).

Four cases with meningiomas anterior to the denticulate ligament were surgically treated. Each operation was performed using intraoperative electrophysiological monitoring *via* somatosensory-evoked potentials (SEPs) and motor-evoked potentials (MEPs). After oral intubation and placement of the electrophysiological monitoring, the head was placed in a Mayfield clamp and the patient was turned into a prone position. After fixation of the Mayfield clamp, the head was positioned in an inclined and elevated position (“Concorde” position). For the present analysis, the distances of the natural gaps between C0 and C1, C1 and C2, and C2 and C3, respectively, were measured using preoperative CT scans and intraoperative lateral x-rays (Figure 1).

#### Case 1

**Figure 2:**
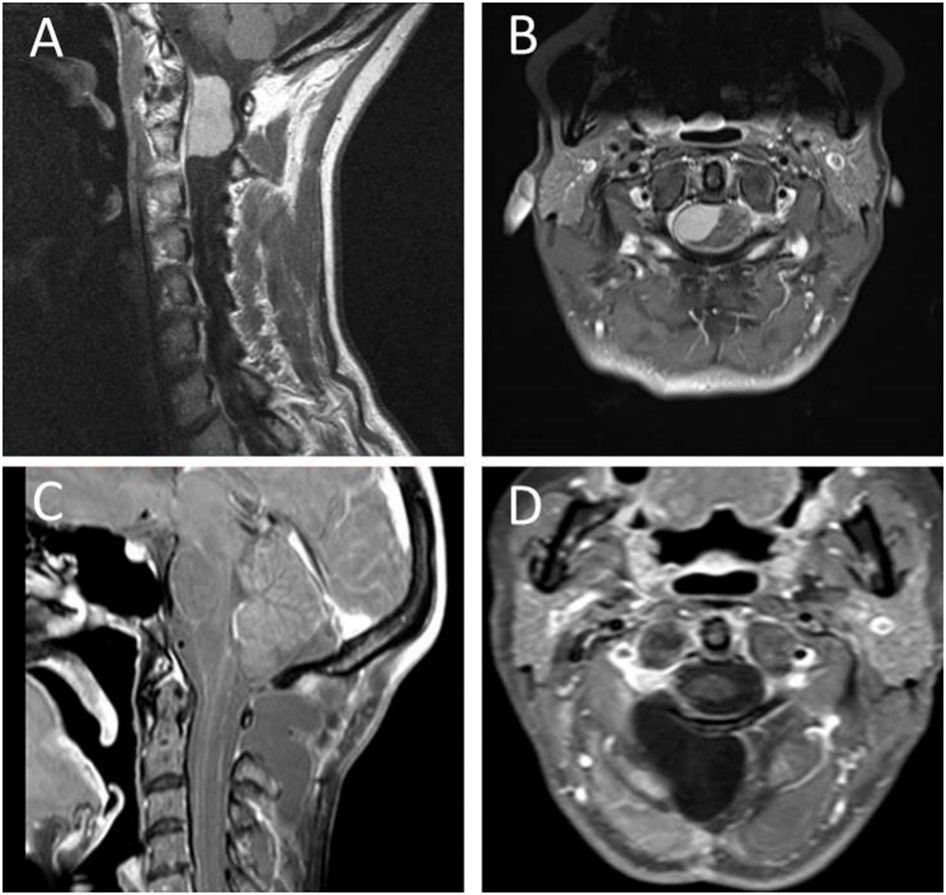
Preoperative sagittal **(A)** and transverse **(B)** T1-weighted contrast enhanced MRI of case 1 depicting an anteriorly located meningioma of the upper cervical spine with cranial extension into the foramen magnum. Postoperative sagittal **(C)** and transverse **(D)** T1-weighed contrast enhanced MRI showing complete resection of the WHO °II meningioma without the need of resecting any boney structures.

Due to a complaint of head and neck pain in a 53-year old female patient, an MRI was performed, which showed an intradural tumor in the CVJ. The tumor reached from the lower clivus down to the lower margin of C2, measuring 24 × 15 × 17 mm. It showed homogeneous contrast enhancement and was located anterolateral to the spinal cord on the right side (Figures 2A,B).

The tumor was approached *via* a midline incision from the occipital protuberance to C3/4. After subperiosteal preparation of the paraspinal muscles, two retractors were placed, which exposed the bony structures from the occiput to C3. The atlantooccipital membrane and the ligamentum flavum were removed between C0 and C1 and C1 and C2, respectively. The dura was separated from the posterior arch of C1 without bone removal. The dura was opened posterolaterally from the foramen magnum to the upper margin of the C2 lamina and was kept open using tuck-up sutures, resulting in a sufficient exposition of the spinal cord, dentate ligament, the exiting dorsal fascicles of C1 and C2, the entry of the vertebral artery, and the posterior surface of the tumor. The microsurgical view was satisfactory above and below the atlas arch covering the entire craniocaudal tumor extension. The posterior surface of the tumor was covered with multiple posterior fascicles that were detached using blunt dissection. Once the transection of the dentate ligament had been performed, the spinal cord could be well mobilized. Due to a rather firm tumor consistency, it had to be sharply divided into small pieces and removed in a piecemeal fashion. After preparation of the adherent medial tumor parts, a broad-based tumor origin was found. The contact point of the tumor was visible anterior to the exit of the C2 fascicles. The dura mater was split and the inner sheath of the dura removed for complete resection of the tumor (Figures 2C,D). The operative time of this surgery was 3 h and 40 min.

#### Case 2

**Figure 3:**
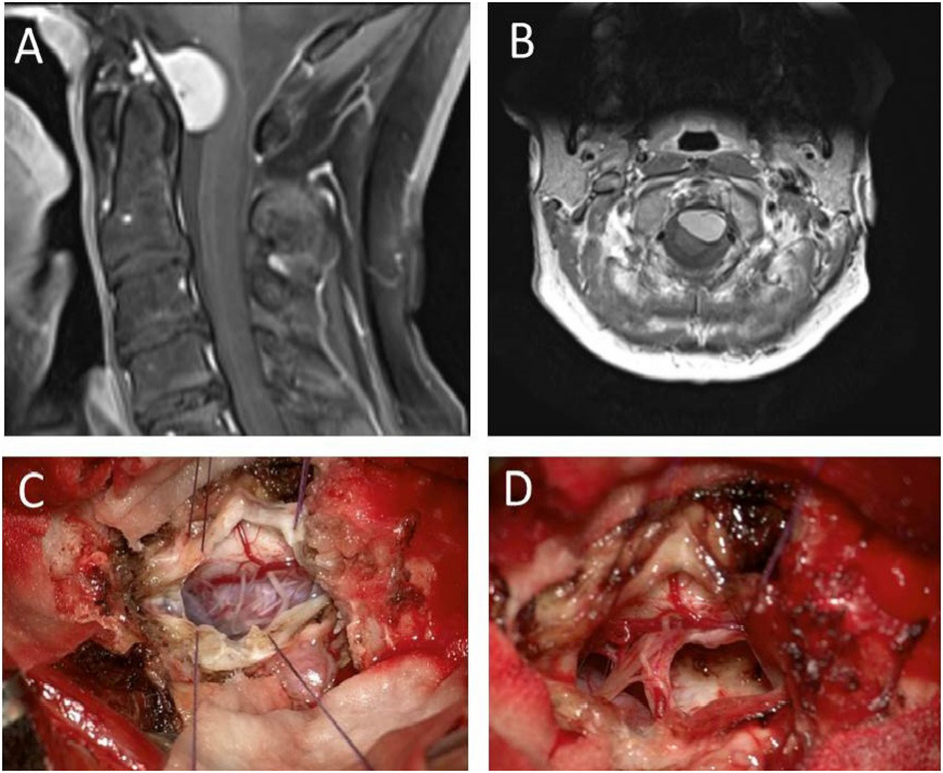
Preoperative sagittal **(A)** and transverse **(B)** images depicting an anteriorly located craniocervical meningioma (case 2) removed through the atlantooccipital space. After dura tuck-up sutures the microsurgical view shows a nicely exposed tumor surface **(C)** without any removal of boney structures. The vascular tumor base was bipolar coagulated and resected in total so that finally a Simpson °II resection was achieved **(D)**.

A 52-year-old female patient was complaining about paresthesia in both arms radiating into all fingers. The symptoms increased with an inclination of the head. Furthermore, she reported a reduction of muscle power and fine motor skills of the right hand. The neuroradiological imaging (including MRI and CT) showed a homogeneously contrast-enhancing tumor with dural tailing in the CVJ, measuring 19 × 17 × 11 mm (diameter 17 mm) and reaching from the lower part of the clivus down to the first third of the odontoid (Figures 3A,B).

The skin incision was performed from the occipital protuberance down to C2. Paraspinal muscles were subperiosteally prepared and two retractors were placed, exposing the bony structures from the occiput to C2. Additional ultrasound navigation was performed, and the anatomical gap through the atlantooccipital membrane was considered wide enough for tumor resection. After dural opening, the microsurgical view showed a well-exposed posterior tumor surface (Figure 3C). Fascicles of the accessory nerve were carefully separated from the medial tumor surface, and the exiting posterior C1 and C2 fascicles were transposed cranially or caudally, respectively. The entry of the vertebral artery was identified. The tumor approached the entry point of the vertebral artery without ensheathing it. A branch of the posterior inferior cerebellar artery was located at the upper tumor margin and was separated from the tumor margin. After bipolar coagulation and incision of the tumor capsule, the tumor mass was of a soft character and could be reduced by simple suction. The tumor base was coagulated and resected. A Simpson °II resection was achieved (Figure 3D). Postoperative CT and MRI scans confirmed the complete resection of the meningioma without removal of any boney structures. The operative time was 3 h and 32 min.

#### Case 3

**Figure 4:**
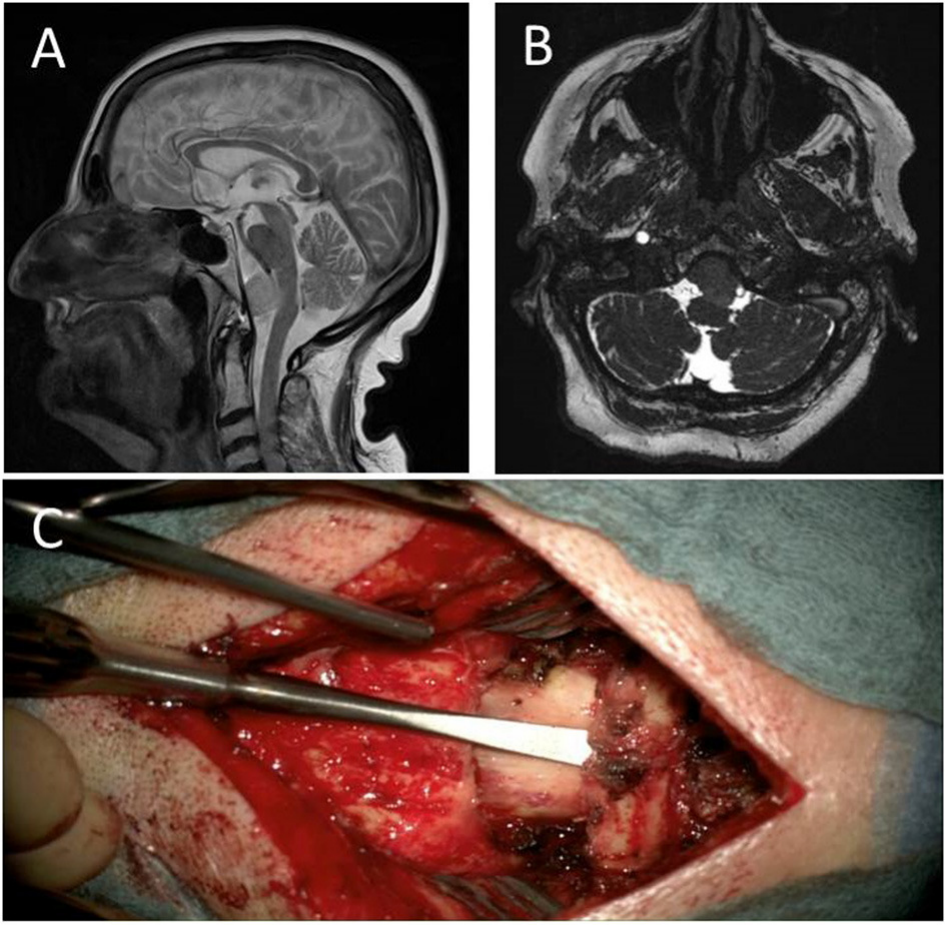
Preoperative sagittal T2-weighted **(A)** MRI of case 3 depicting a foramen magnum and lower clivus meningioma with a cranial extension up to the vertebral junction. Axial 3D-CISS images **(B)** show a sufficiently wide craniocervical cerebrospinal fluid (CSF) space and a bilaterally well distinguishable vertebral artery. After preparation of the paravertebral muscles, an Adson retractor was placed exposing all boney structures from occiput down to C2 **(C)** showing a wide enough space for tumor resection.

Due to right hemispheric headaches, postural vertigo, gait ataxia, and dysmetria for 3 months, an MRI was performed in a 79-year old female patient revealing a meningioma in the CVJ with dural tailing to the lower part of the clivus mediolateral to the right measuring 23 × 20 × 21 mm (diameter 20 mm). Having its origin in the midline on the lower third of the clivus, the tumor dislocated the medulla oblongata posteriorly with a left-sided accentuation (Figures 4A,B).

After preparation of the paravertebral muscles, a retractor was placed exposing all bony structures from the occiput down to the lamina of C2 (Figure 4C). The atlantooccipital membrane was removed and the dura opened with a midline incision between C0 and C1. A lateral extension of the dural incision under microscopic view was performed close to the entry of the vertebral artery. The posterior cord, the denticulate ligament, the spinal fascicles of the accessory nerve, and the entrance of the vertebral artery were clearly visible. The denticulate ligament was incised and an 8.0 suture was placed at the origin of the denticulate ligament, which was used to elevate and slightly torquate the upper cervical cord in order to achieve a broader view of the space anterior of the spinal cord, the tumor surface, and the lower cranial nerves. The tumor was soft but quite highly vascularized. After enucleation with a sharp dissector, suction, and bipolar coagulation of the tumor capsule, the tumor could finally be completely resected. Again, a Simpson °II resection was achieved. The endoscopic view confirmed complete tumor resection without tumor remnants on the clivus, both vertebral arteries and basilar artery. The operative time of this procedure was 2 h and 33 min.

#### Case 4

**Figure 5:**
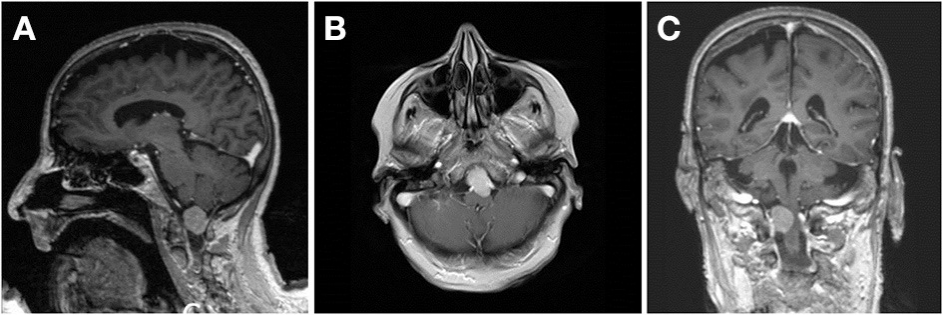
Preoperative sagittal **(A)**, coronary **(B)** and transverse **(C)** contrast-enhanced T1-weighted image of case 4 depicting a lateral craniocervical meningioma growing anterior and posterior from the denticulate ligament.

Due to recurrent syncopic events, a CT scan was performed in a 79-year-old female patient that revealed an incidental tumor in the CVJ. The tumor showed progressive growth in the follow-up MRIs and finally measured 21 × 21 × 18 mm (diameter 18.6 mm) in the CVJ, compressing the medulla oblongata from the anterior right side by 50% and showed calcifications in CT scans (Figures 5A–C). Clinically, the patient suffered from a reduction of fine motor skills of both hands and mild gait ataxia.

After preparation of the paravertebral muscles, a microsurgical lumbar disk retractor (Spine Classics, Aesculap, Tuttlingen, Germany) was placed exposing all bony structures from the foramen magnum to C2. Additional ultrasound navigation was used revealing a space wide enough for an overview of the entire tumor without removal of bony structures of C1 or occiput. The atlantooccipital membrane was incised and folded to the right side. The dura was opened with a midline incision between C0 and C1, including tuck-up sutures. Fibers of the accessory nerve and the exiting C1 and C2 nerve roots were transposed medially respectively cranially or caudally. The entry area of the vertebral artery was identified. At the same time, a very proximal origin of the PICA close to the entry of the vertebral artery was found. The tumor did not surround the vertebral artery or PICA, respectively. On the upper tumor pole, a PICA loop was found. The tumor “content” was soft but not removable by simple suction. Therefore, the tumor capsule and the tumor mass had to be coagulated to carefully be excised in multiple parts. The upper tumor pole was located in the vertebral junction. Favorably, there were no marked tumor capsule adherences to the high vertebral arteries, the low basilar artery, or the PICA loop described above. After coagulation of the tumor origin on the clivus, a resection Simpson °II was achieved. The operative time of this procedure was 2 h and 46 min.

## Results

We treated four patients with intradural lesions located anterior and anterolateral to the neuraxis of the CVJ between September 2017 and June 2019. All included patients were female. Patient ages ranged from 52 to 79 years (mean 65.8 years). Tumor diameters ranged from 17 mm to 21 mm (mean 19.2 mm). All patients underwent surgery *via* a minimally invasive posterior approach using the natural gaps between C0 and C1, C1 and C2, and C2 and 3 without the resection or removal of bony structures. In no case, the surgical approach had to be extended due to intraoperative complications and/or an insufficient microscopic view. Mean operative time using this technique was 3 h and 13 min. Throughout all the procedures, intraoperative electrophysiological monitoring, such as MEPs and SEPs, remained unchanged. All patients underwent complete tumor resection, formally grade °II according to the Simpson classification. Histopathological examinations of the tumors found meningothelial meningioma WHO °I in all cases. Dural closure was performed by a continuous suture technique and an additional collagene/fibrin matrix (TachoSil, Taked, Berlin, Germany). The postoperative clinical course of all patients was uneventful. Postoperative early MRI of Case 1 just showed a subcutaneous CSF collection, which spontaneously disappeared within 6 weeks. Clinically, the patient was without any neurological deficit postoperatively. Patient 2 reported a distinct reduction of her paresthesia in both arms and fine motor deficits three months postoperatively. Patient 3 developed a CSF leak, which was not manageable by lumbar drainage only, so she underwent revision surgery for dural closure, which was free of any perioperative morbidity and with an uneventful postoperative course. Gait ataxia and dysmetria improved after surgery. Patient 4 showed no signs of sensory or motor deficits.

All postoperative MRI scans showed complete tumor resection. During the follow-up time period (range 7–28 months; median 14 months), no tumor recurrences or secondary worsening of symptoms was observed. None of the patients showed postoperative signs of spinal instability.

## Discussion

We report a series of four patients with meningioma in the CVJ operated on *via* a minimally invasive bone-saving approach. Preoperative selection was based on the location of the tumor, the size of the “anatomical gaps” in CT, and plain x-rays and the motion of the upper cervical spine. Intraoperatively, after placement of retractors, ultrasound navigation was used to plan the trajectory and to confirm the accessibility of the tumor prior to opening the dura.

Traditionally, posterior approaches to the cervical spine are used for intraspinal intradural tumors located dorsal or dorsolateral to the spinal cord (8–10). Regarding tumors located anterior or anterolateral to the brainstem or upper spinal cord, the operative strategy and choice of the approach are more complex and challenging. Here, we demonstrate a simple and minimally invasive microsurgical approach to tumors located anterior or anterolateral to the CVJ *via* a posterior approach without the need of removal of any bony structures of the upper spine and/or craniotomy.

Many authors have occupied themselves with the search for the appropriate approaches to lesions located anteriorly in the clivus, the foramen magnum, and the upper cervical spine region. In many cases, far lateral approaches or CVJ approaches with the removal of C1 and/or a craniotomy, including the foramen magnum, were chosen. Even larger, more extensive approaches have been described for this kind of pathology (9–11). One of the most direct and “logical” approaches for ventral lesions would be the transoral approach, which is known to be challenging and with a high risk of CSF leakage, subsequent infections, velopharyngeal function impairment, and a loss of stability. Another disadvantage of this approach is the inadequate exposure of lateral tumor margins and a lack of proximal control of the vertebral artery. The anterior approach described by Banczerowski (12) is usually performed with a complete corpectomy followed by additional stabilization. On the one hand, this approach is well established and more often performed for degenerative spinal pathologies, C1/C2-fractures, and retropharyngeal lesions. This gives a complete view of the anterior aspect of the spinal column and dura. On the other hand, a corpectomy of C2 and the anterior arch of C1 will require a fusion of at least two segments. In contrast to the conventional anterior approach by Banczerowski, the anterolateral and lateral approach described by George (13), mainly used in extradural lesions, and by Yasuda (14) has a reduced bone loss and decreased the risk of the lacerating trachea and/or the esophagus. However, the risk of injury of the accessory nerve or sympathetic chain is increased and the surgical control of vertebral artery or venous plexus hemorrhage is challenging.

Multiple variants for the posterior or posterolateral suboccipital approach have been described, for example, the far lateral and the extreme lateral variants are well established and have a clear indication for large lesions (9–11). In cases of foramen magnum meningioma, almost all of these lesions were resected by George and coworkers *via* a posterolateral approach (far-lateral approach), as detailed in a major publication with more than 100 cases (15). Unfavorable are the time-consuming preparation and the high perioperative morbidity.

In contrast to these approaches, only a few reports on minimally invasive approaches to lesions located anteriorly or anterolaterally can be found in the literature (4–7, 16, 17). The most commonly used approach to the upper cervical spine is the posterior approach through a hemilaminectomy or laminectomy (7). In 1995, Martin et al. described a minimally invasive posterolateral cervical or thoracic approach to vascular malformations and tumors of the ventrolateral aspect of the spinal cord (18). After the multilevel division of the dentate ligament cranial and caudal to the lesion, the authors performed a slight rotation of the spinal cord to approach the lesion. However, this approach is also linked to the brainstem and spinal cord manipulation, risking damage to these sensitive neural structures.

In 2009, Watanabe et al. emphasized the wide space between C1 and C2 (19). In addition, Zozulya et al. described in 2011 that the ratio between the squared spinal cord and dural sac cross-section areas on C1-level is 1:3 on average, which means that 1/3 of the area is occupied by the spinal cord and 2/3 of the area by subarachnoid space. The latter can potentially be used as a trajectory and space for surgical access (11).

In 2016, Eicker et al. accordingly reported that the paramedian spaces between C0–C1 and C1–C2 are 10 mm on average. They optimized the surgical approach by increasing the space through concord/prone positioning of the patient with inclination of the head. They describe an excellent minimally invasive accessibility to small tumors lying in a ventral aspect of the CVJ using a tubular system while avoiding bone removal and extensive muscular mobilization (4). In accordance with Eicker and colleagues, we used a restricted paraspinal subperiosteal posterior approach, but by not using a tubular system. Eicker et al. concluded that the tubular retractor-based bone-sparing approach is restricted only to small lesions anterior to the spinal cord. In our experience, using natural anatomical gaps from the atlantooccipital junction down to C2/C3 for tumor resection without the need for any bony resection, we did not find that the tumor size was the limiting factor. As described in Case 1, tumors exceeding C1 caudally can be treated by a “subsurface” opening of the dura underneath the posterior arch of C1, and tumor preparation can be performed from below and above the posterior arch of the atlas. For tumors extending to the posterior fossa, the clivus angle gives a natural trajectory to get access to these tumors. Dealing with very large tumors, still the conventional anterior, posterior, or lateral approaches to the spinal cord or craniotomy for access to large lesions that reach beyond the foramen magnum will be needed. We propose, however, that the cutting point should not be categorically made at a diameter of 25 mm. Other aspects seem more important, such as a soft texture of the tumor, so that a piecemeal resection or resection of the core of the tumor by simple suction can be performed, appropriate flexibility of the upper cervical spine in order to enlarge and open the natural gaps by the inclination of the head and a not too firm adherence, especially to the vascular structures in this area. We propose that the vertebral junction may be the upper anatomical limit for this approach since microscopic visibility from below along the clivus ends at this point and no control over the vascular structures can be guaranteed if the tumor extends further cranially.

We experienced that apparently “calcified looking” tumors that may be expected when seeing hyperdense lesions on preoperative CT scans are not an absolute exclusion criterion for this minimally invasive approach. We found that, in at least two cases, the lesion appeared hyperdense on preoperative CT, but intraoperatively, a very soft and suctionable tumor was found. However, this approach can always be extended into a conventional hemilaminectomy or laminectomy without the need of changing the OR setting and positioning if the natural space does not seem sufficient or large enough.

Surgical approaches to anteriorly or anterolaterally located tumors of the CVJ are usually complicated and do not rarely involve extensive muscular mobilization and bone removal to get adequate exposure without retraction on neural sensitive structures. For anterior approaches, CSF leakage and infection are the most significant complications. Even in endoscopic anterior surgery, CSF leakage was observed in 18% and meningitis in 5% of patients (20). For lateral and far lateral approaches, significant permanent morbidity has been reported ranging from 0 to 31%, including lower cranial nerve deficits, paralysis, and vascular complications. In addition, temporary morbidity is seen frequently in approximately 25% with typical complications, including transient lower CN palsies, transient hemiparesis, and CSF leaks (21).

The surgical technique presented here is a minimally invasive paraspinal subperiosteal approach to the skull base and upper craniocervical meningiomas without the need for any bone resection. The approach is safe, effective, quick, and easy to learn. In addition, it prevents some known perioperative morbidities using the classic far-lateral or transoral approaches. The operative time between 2.5 and 3.5 h is short and effective and lowers the operative risk of the patient due to general anesthesia. In all operations, the same senior surgeon (T.W.) was present, and due to a learning curve, the operative time improved from case to case.

It has to be considered that the removal of bony structures in the CVJ region does not result in major problems for the patient. Specifically, the removal of the posterior arch of the atlas does not result in biomechanical instability and is frequently performed not only as a surgical approach but also as a treatment of stenosis. However, the search for minimally invasive strategies with as little change of the natural physiological texture is the natural course of surgical research. The issue is similar to the development of disk surgery where surgical approaches have become less and less invasive over the decades, bony structures have been increasingly saved, and eventually, endoscopic disk surgery has been developed without any bone removal at all. There is certainly no evidence that the approach we propose in this report is of long-term clinical advantage. However, it is simple and keeps the structures of the spine intact.

In this series, we observed that postoperative local pain was extremely low, possibly due to the fact that no bony structure was removed, and there was only very little demand for analgesics. Given that no hemilaminectomy or laminectomy was performed and the lamina stays intact, no “sinking effect” of the paraspinal muscles was observed. Considering the long-term results, there is certainly no risk of postoperative instability if all bony structures stay untouched. In summary, the least possible alteration of biomechanical conditions is achieved.

## Conclusion

A minimally invasive posterior cervical approach can be used to reach ventrally located tumors of the CVJ without the need for resecting any bony structures and without the need for further technical equipment other than the usual microsurgical OR setting. Natural gaps between the atlantooccipital junction, C1/2, and C2/3, may be used to approach the pathology. Patients with tumors not extending higher than the vertebral junction and a fairly mobile C-spine with sufficient width of the posterior bony gaps may be good candidates for this approach. High perioperative morbidities due to large musculoskeletal preparation, infections, CSF leak, or postoperative stabilization are minimalized in comparison to the classical anterior or extensive posterior approaches.

## Data Availability Statement

The original contributions generated for the study are included in the article/supplementary material, further inquiries can be directed to the corresponding author/s.

## Ethics Statement

Ethical review and approval was not required for the study on human participants in accordance with the local legislation and institutional requirements. The patients/participants provided their written informed consent to participate in this study.

## Author Contributions

NL and TW contributed to conception and design of the study. AK and JW organized patients data(base) for case series report. NL wrote the first draft of the manuscript. NL, AK, JW, R-IE, and TW wrote sections of the manuscript. All authors contributed to manuscript revision, read, and approved the submitted version.

## Conflict of Interest

TW received lecture fees from Medtronic Navigation and Raumedic. The remaining authors declare that the research was conducted in the absence of any commercial or financial relationships that could be construed as a potential conflict of interest.

## Publisher's Note

All claims expressed in this article are solely those of the authors and do not necessarily represent those of their affiliated organizations, or those of the publisher, the editors and the reviewers. Any product that may be evaluated in this article, or claim that may be made by its manufacturer, is not guaranteed or endorsed by the publisher.
